# Association of Skull Type, Diet, and Chronic Gingivostomatitis with Tooth Resorption in Cats Receiving Dental Treatment

**DOI:** 10.3390/ani16010135

**Published:** 2026-01-03

**Authors:** Pitak Anusorn, Chakkarin Satthathum, Pollawat Jariyarangsrirattana, Emmita Mongkholdej, Doungnapa Onnom, Naris Thengchaisri

**Affiliations:** 1 Surgery Unit, Faculty of Veterinary Medicine, Kasetsart University Veterinary Teaching Hospital, Kasetsart University, 50 Ngamwongwan Road, Donmueang, Bangkok 10900, Thailand; docboxbox@gmail.com (P.A.); fvetcrs@ku.ac.th (C.S.); fvetpwj@ku.ac.th (P.J.); emmita.m@ku.th (E.M.); onnom_d@hotmail.com (D.O.); 2 Department of Companion Animal Clinical Sciences, Faculty of Veterinary Medicine, Kasetsart University, 50 Ngamwongwan Road, Donmueang, Bangkok 10900, Thailand

**Keywords:** cats, tooth resorption, dental radiography, diet, gingivostomatitis, skull type

## Abstract

Tooth resorption (TR) is a common and painful dental disease in cats. The contributions of skull type, diet, and chronic gingivostomatitis (CGS) to its development remain unclear. We retrospectively reviewed 166 cats with TR confirmed radiographically to evaluate these associations. Brachycephalic cats (*N* = 33) were significantly younger than non-brachycephalic cats (7.1 ± 2.6 vs. 8.7 ± 3.8 years, *p* = 0.026) and had a higher prevalence of advanced Stage 4 TR lesions (*p* = 0.018). There was no significant difference between two groups of cats in sex distribution, diet type or wet food consumption. CGS occurred more often in non-brachycephalic cats (57.9% vs. 21.2%, *p* < 0.001) but was not associated with TR severity. In both skull groups, mandibular premolars and molars were most commonly affected (*p* < 0.01). Cats with owner-reported premium diets had more Stage 4 lesions (*p* = 0.013), particularly in non-brachycephalic cats but not in brachycephalic cats. These findings suggest that TR severity is associated with younger age and advanced lesions in brachycephalic breeds, as well as diet-related differences in non-brachycephalic cats. Further studies are warranted to evaluate early dental screening and targeted nutritional strategies to mitigate the progression of tooth resorption in cats.

## Simple Summary

Tooth resorption is a common and painful dental condition in cats. Its causes, particularly in young brachycephalic cats, are not well understood. In this study of 166 cats, we evaluated the links between skull type, diet, and chronic oral inflammation and how they are related to disease severity. Brachycephalic cats (e.g., Persians) were younger but had already developed advanced lesions. In contrast, non-brachycephalic cats more frequently had chronic gingivostomatitis, which was not associated with increased tooth resorption. Cats fed premium diets showed a higher prevalence of severe lesions. Overall, these findings suggest that skull type and cat diet may have a stronger influence on tooth resorption. Although a direct cause cannot be proven, more research is needed to determine whether early dental checks and special diets can slow tooth resorption in cats.

## 1. Introduction

Feline oral diseases are common and clinically important in domestic cats. These include tooth resorption (TR), chronic gingivostomatitis (CGS), and periodontitis, with prevalence reported to vary across geographic areas and study populations [1,2,3,4,5]. TR is a progressive dental condition in which odontoclast-like cells resorb dental hard tissues. This process usually begins at the tooth roots and can extend into the crown [6]. The prevalence of TR increases with age in cats [2,6]. Archaeological evidence indicates that TR occurred in cats as early as the 13th and 14th centuries [7]. Accurate diagnosis is essential. Cone-beam computed tomography (CBCT) provides greater accuracy than conventional dental radiography, which remains widely used due to its practicality and accessibility [8,9]. Emerging AI-assisted techniques may further enhance detection [10]. Affected teeth are classified into five stages and three radiographic types according to the American Veterinary Dental College (AVDC) system [11].

The pathogenesis of TR is multifactorial, involving cytokine-mediated odontoclastic activity [12], adhesion molecule expression [13], and matrix metalloproteinase-9 (MMP-9) expression [14]. Chronic oral inflammation and microbial imbalance also play important roles. Alterations in the oral microbiota can lead to dysbiosis and local inflammation, contributing to the development of feline TR (also known as FORL) [15]. Elevated inflammatory cytokines may contribute to odontoclast activation [16,17], and in cats with CGS, inflammation-driven shifts in the oral microbiota correlate with disease severity [18]. Breed-related craniofacial variations may predispose cats to TR [19], potentially interacting with local inflammatory mechanisms. These observations are further supported by studies showing that gingiva-derived mesenchymal stromal cells from cats with TR exhibit impaired proliferation, increased apoptosis and senescence, and altered cytokine expression, indicative of compromised tissue homeostasis [20]. Additionally, mast cell- and complement-mediated alveolar bone pathology may also contribute to disease progression [21]. Despite identified mechanism, the exact causes of TR remain unclear [22]. TR pathogenesis likely involves interaction among local inflammation, immune signaling, and cellular dysfunction.

Systemic factors, reflected by altered creatinine and globulin levels, have been associated with TR [23]. Low serum 25-OH-D (vitamin D_3_) is associated with an increased prevalence of multiple lesions [24], whereas excess dietary vitamin D may be the long-sought cause of multiple TR in domestic cats [25], highlighting that both deficiency and excess may influence dental health. Probiotics, though not directly studied in TR, modulate the feline oral microbiota and may indirectly support oral health [26], whereas diet—particularly dry kibble—reduces plaque and gingival inflammation, supporting periodontal health and potentially lowering TR risk [27]. Clinically, tooth extraction remains the treatment of choice, particularly in cases of pain, severe lesions, or functional impairment [6,28]. However, understanding the mechanisms driving disease progression remains essential for improving long-term outcomes. Therefore, the objective of this study was to retrospectively determine how skull type, diet, and CGS are associated with the severity of TR in cats. Relevant demographic and clinical factors recorded from the hospital database were also analyzed to identify potential contributors to TR progression.

## 2. Materials and Methods

### 2.1. Study Design and Ethical Approval

This retrospective observational study was conducted using clinical records from cat patients diagnosed with TR at the Kasetsart University Veterinary Teaching Hospital (KUVTH), Bangkok, Thailand. Clinical records from March 2021 to August 2025 were retrospectively reviewed. Ethical approval was obtained from the Kasetsart University Institutional Animal Care and Use Committee (IACUC; approval number #ACKU68-VET-105) and the Ethical Review Board of the Office of the National Research Council of Thailand (NRCT license U1-07457-2561). Written informed consent was obtained from all owners before inclusion. All procedures complied with institutional animal welfare regulations and were conducted in accordance with the Kasetsart University guidelines for animal care and use.

### 2.2. Study Population

**Figure 1:**
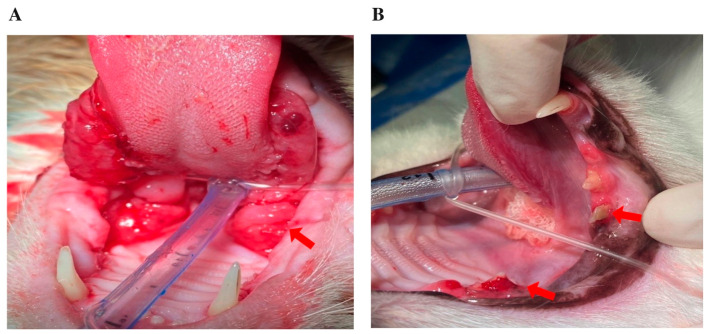
Representative intraoral images of cats under general anesthesia, depicting: (**A**) severe, bilateral ulcerative lesions affecting the palatoglossal arches and caudal oral mucosa, consistent with chronic gingivostomatitis (arrows); (**B**) fractured and discolored teeth, with gingival inflammation suggestive of tooth resorption (arrows).

A total of 166 domestic cats diagnosed with TR during dental evaluations were included in this study. Cats were classified into two groups according to skull morphology. The brachycephalic group (*N* = 33) consisted of breeds such as Persian, Exotic Shorthair, and British Shorthair, characterized by a shortened facial structure and a broad skull base. The non-brachycephalic group (*N* = 133) included domestic shorthairs, Siamese, and other breeds exhibiting either mesocephalic or dolichocephalic skull types. Inclusion required complete clinical and dental records (Figure 1), a documented dietary history, and high-quality diagnostic full-mouth intraoral radiographs. Exclusion criteria included incomplete records, poor-quality radiographs, the presence of systemic illnesses known to affect bone metabolism (such as primary hyperparathyroidism or chronic kidney disease), or a history of dental extractions that could interfere with lesion assessment.

### 2.3. Data Collection and Radiographic Evaluation

**Figure 2:**
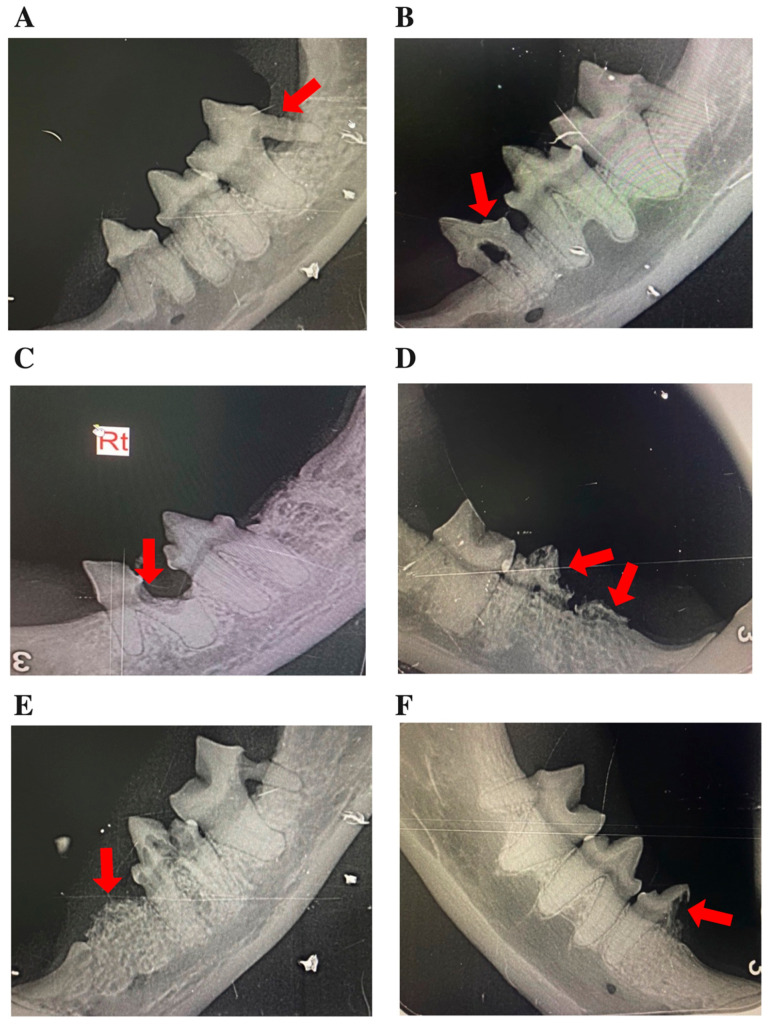
Representative intraoral radiographs of mandibular premolars and molars in cats, depicting: (**A**–**E**) tooth resorption (TR) stages 1–5, illustrating the progressive loss of dental hard tissue, as indicated by arrows; and (**F**) a mixed lesion exhibiting features of both Type 1 and Type 2 TR (arrow). In panel C, “Rt” denotes the right side.

Data were retrieved from the hospital’s electronic medical record system, including demographic information (age, sex, breed) and dental findings. Full-mouth intraoral radiographs were obtained under general anesthesia using the PORT-X IV Portable X-ray system (Genoray, Seongnam-si, South Korea) and the CR 7 Vet Image Plate Scanner (iM3 Dental Limited, Duleek, Ireland), following standardized hospital protocols. Lesions were classified by type (1–3) and stage (1–5) according to AVDC criteria [11]. The location of affected teeth was recorded, distinguishing maxillary versus mandibular involvement and identifying the specific tooth group (incisor, canine, premolar, or molar). The presence of CGS was determined from clinical examination and intraoral inspection (Figure 1). Representative clinical and radiographic images of CGS, periodontal disease, and TR lesions at different stages are presented in Figure 2. All radiographs were independently reviewed by three trained clinicians, and any discrepancies were resolved by consensus. Formal inter-observer reliability testing was not performed and is acknowledged as a limitation. All evaluators followed standardized scoring criteria to ensure consistency and reduce variability in lesion classification.

### 2.4. Dietary Assessment

Dietary histories were obtained from owners during admission interviews. Diets were classified into two categories premium and commercial based on a pragmatic distinction rather than detailed quantitative nutrient analysis. Premium diets were defined as well-known international brands containing higher-quality protein sources, whereas commercial diets comprised locally produced or OEM products with lower-quality protein sources and less established branding. Wet food consumption was recorded as a binary variable (yes/no).

For cats receiving mixed diets, classification was based on the predominant daily diet reported by owners when exact proportions were unavailable. This approach may have introduced recall bias, which is acknowledged as a limitation of the study.

### 2.5. Statistical Analysis

Statistical analyses were performed using GraphPad Prism version 9 (GraphPad Software, Boston, MA, USA) and Stata version 12.1 (StataCorp, College Station, TX, USA). Descriptive statistics were used to summarize the clinical and demographic characteristics of feline patients. Number of teeth affected with different stages of TR was presented as the mean ± standard deviation and compared between two different skull types (brachycephalic vs. non-brachycephalic), two different diets (premium vs. commercial diets), and between cats with or without CGS using Student’s *t*-tests. Categorical variables (sex, CGS lesions, diet type, and wet food feeding) were analyzed using Fisher’s exact test or the χ^2^ test, as appropriate. A *p*-value < 0.05 was considered statistically significant.

## 3. Results

In this retrospective study of 166 cats with TR, brachycephalic cats (*N* = 33) were significantly younger than non-brachycephalic cats (7.1 ± 2.6 vs. 8.7 ± 3.8 years, *p* = 0.026) (Table 1) and had more advanced Stage 4 TR lesions (*p* = 0.018). CGS was significantly more prevalent in non-brachycephalic cats (57.9% vs. 21.2%, *p* < 0.001). No significant differences were observed between the groups in sex distribution and lesion types. Other TR stages, diet type, and wet food consumption also did not differ significantly (Table 1).

The distribution of teeth affected by TR was assessed in the brachycephalic and non-brachycephalic cats (Table 2). In brachycephalic cats, the mandibular molars were significantly more affected than maxillary molars (*p* < 0.01). In non-brachycephalic cats, the mandibular premolars and molars of mandible were more often affected than those in the maxilla (*p* < 0.01). No statistically significant differences were detected between skull types for individual tooth categories or jaw regions, indicating a consistent mandibular predominance regardless of craniofacial morphology.

When comparing TR severity between cats fed premium and commercial diets (Table 3), cats consuming premium diets had a significantly higher proportion of Stage 4 lesions (*p* = 0.013), whereas no significant differences were observed for Stages 1, 2, 3, or 5. This finding suggests that advanced TR tended to occur more frequently in cats fed premium diets, although overall lesion distribution remained similar between diet groups.

The severity of TR was further analyzed in brachycephalic cats according to diet type (premium vs. commercial; Table 4). No statistically significant differences were observed at any lesion stage. There was a trend for Stage 3 lesions to be more frequent in cats fed commercial diets (7.8% vs. 4.2%, *p* = 0.077), whereas Stage 4 lesions tended to be slightly higher in cats fed premium diets (6.3% vs. 3.9%, *p* = 0.256). Overall, these trends did not reach statistical significance, suggesting that diet type was not strongly associated with TR severity in brachycephalic cats.

In contrast, within the non-brachycephalic group, cats fed premium diets exhibited a significantly higher number of Stage 4 lesions compared with those fed commercial diets (*p* = 0.020), suggesting a greater prevalence of advanced disease in this group (Table 5). No significant differences were observed between diet types for Stages 1, 2, 3, or 5, indicating that the earlier stages of TR were similarly distributed regardless of diet.

Additionally, when comparing cats with and without CGS, no significant differences were observed in the number of teeth affected at any stage of TR (Table 6). Stage 1 lesions tended to be more common in cats without CGS (*p* = 0.053), but this trend did not reach statistical significance, suggesting that CGS was not associated with either the severity or prevalence of TR.

## 4. Discussion

Skull morphology influenced TR severity in our study, with notable differences observed between brachycephalic and non-brachycephalic cats. While the prevalence of TR generally increases with age [2,6,29], we observed that even younger brachycephalic cats may develop disproportionately advanced (Stage 4) lesions. The brachycephalic conformation of Persian and Exotic cats is associated with unique oral and dental features that could predispose them to dental diseases, including tooth resorption [19]. However, other evidence indicates that the Exotic-Persian group may have a lower TR prevalence compared to house cats, whereas Cornish Rex, European, and Ragdoll cats show higher susceptibility [29]. These divergent findings indicate the variability in breed-related risk and emphasize the need for further research to elucidate the roles of craniofacial structure and genetics in TR progression. Regarding lesion distribution, mandibular premolars and molars were most frequently affected, consistent with previous reports identifying the mandibular fourth premolars as commonly involved [2,30].

CGS was more prevalent in non-brachycephalic cats in our cohort; however, it was not associated with greater TR severity, suggesting that oral inflammation alone does not fully account for lesion development. TR pathogenesis is multifactorial, with cytokine-mediated odontoclastic activity playing a central role [12], and dysbiosis of the oral microbiota potentially contributing to local inflammation [15]. In affected cats, pro-inflammatory cytokines are elevated in saliva [16], while both pro- and anti-inflammatory cytokines are upregulated in gingival tissues [17], reflecting an inflammatory environment that may promote odontoclast activation. Locally produced 1,25-dihydroxyvitamin D, potentially enhanced by inflammation [31], likely drives odontoclast activity in TR, independent of serum vitamin D levels, as evidenced by upregulation of vitamin D receptor and target genes [32]. Abnormal blood parameters, including electrolyte imbalances, may reflect systemic effects associated with tooth resorption [33]. Overall, severe tooth resorption in cats arises from a complex interaction of microbial, immune, and systemic factors, with inflammation necessary but not sufficient on its own.

**Figure 3:**
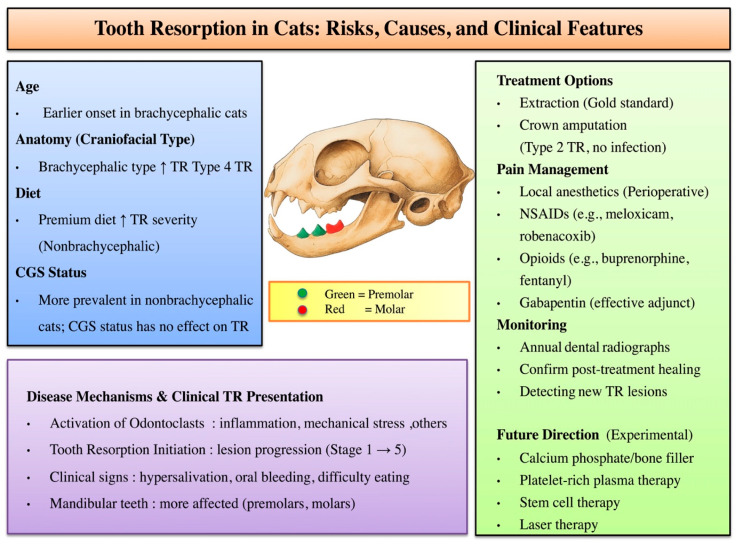
Conceptual framework of TR illustrating major risk factors (age, skull type, and diet), lesion distribution, and clinical outcomes. Mandibular premolars (green) and molars (red) are most frequently affected. Proposed pathogenic pathways of TR development are presented, emphasizing odontoclast activation and the role of inflammatory mediators. Current treatment options and pain management strategies are outlined. Further development of targeted therapies and improved monitoring approaches is needed for TR. Upward arrows indicate an increase in risk or disease progression.

Diet type, particularly the distinction between dry kibble and wet food, is thought to play an important role in feline dental health. Dry kibble can reduce plaque accumulation and gingival inflammation through a mechanical cleaning effect, thereby potentially lowering the risk of oral diseases, including tooth resorption [27]. In our study, the comparison focused on diet quality (premium vs. commercial) rather than form. Non-brachycephalic cats fed premium diets exhibited more advanced TR lesions (Figure 3). This suggests that dietary composition and nutrient profile, rather than food texture alone, may influence the progression of TR lesion. Previous studies have indicated that cats with multiple TR lesions may have lower serum vitamin D_3_ concentrations, potentially promoting odontoclastic activity, even when consuming lifelong premium dry diets [24].

Many previous studies on TR have focused on cats from Western countries, whereas our study provides insights from an Asian cohort, where breed distributions and feeding practices may differ. These regional variations could influence the development and progression of TR through both genetic and environmental factors, highlighting the need for broader, population-based studies to better understand the disease’s multifactorial nature. In the present study, brachycephalic cats appear predisposed to faster TR progression, and diet may further exacerbate TR severity. Although CGS was more common in non-brachycephalic cats, it did not worsen TR. Figure 3 illustrates how inflammatory, dietary, and anatomical risk factors may converge to accelerate TR lesion development from Stage 1 through Stage 5. Current dental management focuses on extraction or crown amputation for Type 2 lesions, along with pain control using local anesthetics, NSAIDs, opioids, or gabapentin. Regular dental radiographs are essential to detect new TR lesions and confirm healing. Experimental treatments, such as calcium phosphate/bone fillers, platelet-rich plasma, stem cell therapy, and laser treatment, may offer future options for TR management (Figure 3).

## 5. Limitations

This study has several limitations. Its retrospective design limits causal inference and control for confounding factors, including age, which was not adjusted for and may partially explain the higher prevalence of CGS in non-brachycephalic cats. Dietary assessment relied on owner reports and product labeling, precluding attribution of effects to specific nutrients such as vitamin D. Radiographic scoring lacked formal inter-observer reliability testing. Breed-specific genetic contributions to TR were not investigated, and outcomes were not compared between screened and unscreened cats. Moreover, the impact of home diet and home dental care were not evaluated. Despite these limitations, consistent trends across skull types and dietary groups support the robustness of our findings. Future prospective studies with larger, balanced cohorts should use age-adjusted, multivariable analyses. The effects of dental care, genetics, home diet, and systemic factors on severity of TR should also be evaluated.

## 6. Conclusions

This retrospective study revealed that brachycephalic cats develop more advanced TR at a relatively younger age compared to non-brachycephalic cats. The mandibular premolars and molars are the teeth most frequently affected, suggesting mandibular teeth as particularly susceptible sites for TR development. Although chronic gingivostomatitis was more prevalent in non-brachycephalic cats, it was not associated with TR severity, suggesting that CGS may not play a major role in TR progression. Premium diets were associated with greater TR severity in non-brachycephalic cats, although this relationship should be interpreted with caution given the retrospective design and reliance on owner-reported dietary information. Overall, the present findings support the roles of skull type, tooth location, and diet as potential contributors to TR development and progression in cats. Further studies are needed to evaluate the benefits of early dental screening in at-risk cats, as well as the potential role of specific nutritional interventions in reducing TR progression.

## Figures and Tables

**Table 1:** Demographics of brachycephalic and non-brachycephalic cats with tooth resorption (TR).

Parameters	TR in Brachycephalic Cats	TR in Non-Brachycephalic Cats	*p*-Value
No. of patient	33	133	-
Age (years)	7.1 ± 2.6	8.7 ± 3.8	0.026
Gender			
Male	15 (45.5%)	62 (46.6%)	
Female	18 (54.5%)	71 (53.4%)	
TR lesion			
Type 1 (%)	9.7 ± 7.4	10.0 ± 7.4	0.821
Type 2 (%)	6.5 ± 8.0	6.1 ± 8.1	0.799
Type 3 (%)	1.9 ± 3.3	1.5 ± 2.1	0.429
%TR lesion			
Stage 1 (%)	0.0 ± 0.0	0.2 ± 0.7	0.180
Stage 2 (%)	2.5 ± 3.6	2.4 ± 3.3	0.891
Stage 3 (%)	5.9 ± 5.8	6.5 ± 5.8	0.557
Stage 4 (%)	5.3 ± 6.1	3.4 ± 3.5	0.018
Stage 5 (%)	4.5 ± 6.0	5.2 ± 7.7	0.586
CGS lesion			
Yes	7 (21.2%)	77 (57.9%)	<0.001
No	26 (78.8%)	56 (42.1%)	
Type of diet			
Commercial	15 (45.5%)	65 (48.9%)	0.725
Premium	18 (54.5%)	68 (51.1%)	
Fed on wet food			
Yes	16 (48.5%)	62 (46.6%)	0.847
No	17 (51.5%)	71 (53.4%)	

**Table 2:** Comparison of the number of teeth affected by tooth resorption (TR) in the maxilla and mandible of brachycephalic and non-brachycephalic cats.

TR Location	Brachycephalic Cats (*N* = 33)		Non-Brachycephalic Cats (*N* = 133)	
	Maxilla	Mandible	Maxilla	Mandible
Incisor	0.2 ± 0.7	0.1 ± 0.7	0.2 ± 1.0	0.5 ± 1.4
Canine	0.2 ± 0.5	0.3 ± 0.7	0.2 ± 0.5	0.2 ± 0.5
Premolar	1.3 ± 1.7	2.2 ± 1.3	1.2 ± 1.4	2.0 ± 1.3 ^##^
Molar	0.1 ± 0.3	1.2 ± 0.8 **	0.1 ± 0.3	1.2 ± 0.8 ^##^

Note: ** *p* < 0.01 vs. maxilla (within the brachycephalic group); ^##^ *p* < 0.01 vs. maxilla (within the non-brachycephalic group).

**Table 3:** Comparison of the number of teeth affected by different stages of tooth resorption (TR) in the maxilla and mandible in cats fed commercial or premium diets.

TR Stages	TR in Cats Fed Premium Diets (*N* = 86)	TR in Cats Fed Commercial Diets (*N* = 80)	*p*-Value
Stage 1 (%)	0.1 ± 0.6	0.2 ± 0.7	0.631
Stage 2 (%)	2.4 ± 3.2	2.3 ± 3.5	0.861
Stage 3 (%)	6.1 ± 5.2	6.7 ± 6.3	0.548
Stage 4 (%)	4.5 ± 4.6	2.9 ± 3.5	0.013
Stage 5 (%)	5.5 ± 7.0	4.7 ± 7.8	0.509

**Table 4:** Comparison of the number of teeth affected by different stages of tooth resorption (TR) in the maxilla and mandible in brachycephalic cats fed premium or commercial diets. NS, not significant.

TR Stages	Brachycephalic Cats Fed Premium Diets (*N* = 18)	Brachycephalic Cats Fed Commercial Diets (*N* = 15)	*p*-Value
Stage 1 (%)	0.0 ± 0.0	0.0 ± 0.0	NS
Stage 2 (%)	2.3 ± 4.0	2.7 ± 3.3	0.729
Stage 3 (%)	4.2 ± 3.7	7.9 ± 7.3	0.065
Stage 4 (%)	6.3 ± 6.9	4.2 ± 4.8	0.334
Stage 5 (%)	4.2 ± 5.3	4.8 ± 6.9	0.769

**Table 5:** Comparison of the number of teeth affected by different stages of tooth resorption (TR) in the maxilla and mandible in non-brachycephalic cats fed premium or commercial diets.

TR Stages	Non-Brachycephalic Cats Fed Premium Diets (*N* = 68)	Non-Brachycephalic Cats Fed Commercial Diets (*N* = 65)	*p*-Value
Stage 1 (%)	0.1 ± 0.6	0.2 ± 0.8	0.657
Stage 2 (%)	2.5 ± 3.0	2.3 ± 3.5	0.698
Stage 3 (%)	6.7 ± 5.4	6.4 ± 6.1	0.789
Stage 4 (%)	4.1 ± 3.7	2.6 ± 3.1	0.016
Stage 5 (%)	5.8 ± 7.4	4.7 ± 8.1	0.405

**Table 6:** Comparison of the number of teeth affected by different stages of tooth resorption (TR) in the maxilla and mandible of cats with and without chronic gingivostomatitis (CGS).

TR Stages	TR in Cats with CGS (*N* = 84)	TR in Cats Without CGS (*N* = 82)	*p*-Value
Stage 1 (%)	0.0 ± 0.3	0.2 ± 0.8	0.053
Stage 2 (%)	2.5 ± 3.6	2.3 ± 3.0	0.691
Stage 3 (%)	6.5 ± 5.4	6.3 ± 6.2	0.870
Stage 4 (%)	3.4 ± 3.6	4.2 ± 4.7	0.239
Stage 5 (%)	5.2 ± 8.1	4.9 ± 6.7	0.775

## Data Availability

The datasets generated or analyzed during this study are available from the corresponding author upon reasonable request.

## Abbreviations

The following abbreviations are used in this manuscript:

TR	Tooth Resorption
CGS	Chronic Gingivostomatitis
FORL	Feline Odontoclastic Resorptive Lesion
CBCT	Cone-Beam Computed Tomography
MMP-9	Matrix Metalloproteinase-9
AVDC	American Veterinary Dental College
NSAIDs	Nonsteroidal Anti-Inflammatory Drugs
